# A Comprehensive Review of the Neurological Manifestations of Celiac Disease and Its Treatment

**DOI:** 10.3390/diseases10040111

**Published:** 2022-11-21

**Authors:** Dhir Gala, Shelbie Scharf, Megan Kudlak, Christian Green, Faisal Khowaja, Mili Shah, Vikash Kumar, Gautam Ullal

**Affiliations:** 1 American University of the Caribbean School of Medicine, 1 University Drive at Jordan Dr, Cupecoy, Sint Maarten, The Netherlands; 2 Department of Internal Medicine, The Brooklyn Hospital Center, 121 DeKalb Ave, Brooklyn, NY 11201, USA; 3 Department of Neuroscience, American University of the Caribbean School of Medicine, 1 University Drive at Jordan Dr, Cupecoy, Sint Maarten, The Netherlands

**Keywords:** celiac disease, neurological manifestations, epilepsy, headache, neuropathy

## Abstract

Celiac disease (CD) is a common chronic inflammatory disorder occurring in genetically predisposed individuals secondary to gluten ingestion. CD usually presents with gastrointestinal symptoms such as pain, bloating, flatulence, and constipation or diarrhea. However, individuals can present in a nonclassical manner with only extraintestinal symptoms. The neurological manifestations of CD include ataxia, cognitive impairment, epilepsy, headache, and neuropathy. A lifelong gluten-free diet is the current recommended treatment for CD. This review discusses the relevant neurological manifestations associated with CD and the novel therapeutics. Further research is required to get a better understanding of the underlying pathophysiology of the neurological manifestations associated with CD. Clinicians should keep CD in the differential diagnosis in individuals presenting with neurological dysfunction of unknown cause.

## 1. Celiac Disease

### 1.1. Introduction

Celiac Disease (CD) is one of the most common genetically based diseases across European countries and the United States. It is a systemic immune mediated disorder, targeted to the small intestine, that occurs secondary to ingestion of gluten, a protein found in wheat, rye, and barley [1]. Due to the high prevalence of asymptomatic cases, it is difficult to determine the exact prevalence of CD [2,3]. Typical symptoms vary depending upon whether the patient is a child or an adult. They generally include gastrointestinal (GI) discomfort, abdominal pain, bloating, flatulence, constipation, diarrhea, vomiting, and anorexia [4]. Other symptoms may include chronic fatigue, nutrient deficiencies, poor growth, and failure to thrive [5]. Additionally, dermatological symptoms of CD may include rash or ulceration [6]. Most often CD is first detected between ages 6–18 months, as foods containing prolamines, the most common gluten trigger, are generally introduced into the diet at this time [2,7]. Atypical forms are usually diagnosed in older children and adults, as those symptoms are more extra-intestinal and do not present with the classical symptoms of malabsorption [2].

CD is a multifactorial disorder, requiring both an environmental trigger and genetic predisposition for development. An increased number of infections during childhood, especially from Reovirus or Retrovirus could also predispose one to develop CD [8].

### 1.2. Pathophysiology

Those at the highest risk for developing CD generally have a strong family history of the disease. The key genetic components include human leukocyte antigens (HLA) molecules: HLA-DQ2 and HLA-DQ8. Over 90% of patients with diagnosed CD express HLA-DQ2, and patients homozygous with HLA-DQ2 have the most increased risk of developing the disease [3]. These HLA molecules play an especially important part in the pathogenesis of CD and are involved in an inappropriate adaptive immune response to peptides from gluten molecules. Prolamine is a hydrophobic, alcohol soluble, storage form of cereal grain [9]. Its dietary form is called gluten and causes initiation of immune response in CD [10]. HLA-DQ2 or HLA-DQ8 present epitopes from prolamine producing a CD4+ T-lymphocyte predominant response. In a study Novel avian single-chain fragment variable (scFv) showed promising results against peptic-tryptic digest of gliadin (Pt-G) and can potentially be used as a treatment for CD [10].

**Figure 1:**
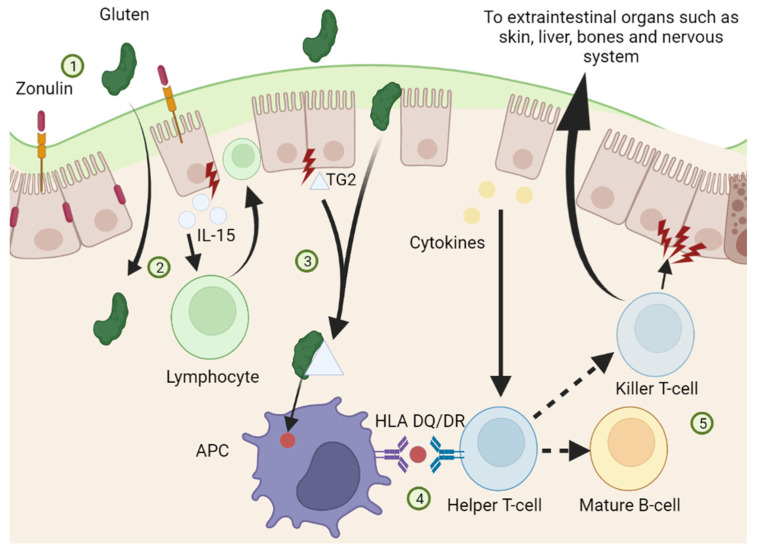
Pathophysiology of Celiac Disease. Gluten is digested, which causes enterocytes to release zonulin proteins. Zonulin loosens the tight junctions between the enterocytes, allowing the gluten particles to translocate into the lamina propria (1). Once in the lamina propria, gluten particles cause inflammatory cells to release cytokines, including IL-15. IL-15 activates lymphocytes, which damage the enterocytes (2). Damaged enterocytes release an enzyme called transglutaminase 2 (TG2) that works to break down gluten particles. These gluten particles are transported via TG2 to APC’s (3). APC’s present the gluten particles to helper T cells. Inflammatory cytokines from damaged enterocytes also work to activate helper T-cells, which go on to form killer T-cells and to stimulate mature B-cells that work in part to further damage the enterocytes (4). Some of the Killer T-cells translocate to other regions of the body, causing manifestations of the musculoskeletal, neurologic, hepatic and endocrine systems (5).

Another gluten, called gliadin is sparingly digested by pancreatic and gastric enzymes, interacts with enterocytes to disassemble the tight junctions between cells. Disruption of the tight junctions allows for zonulin to be upregulated, increasing gut permeability. With an increase in gut permeability, gluten molecules such as gliadin can translocate into the lamina propria and activate the T-lymphocytes located there. Transglutaminase-2 (TG2) is thought to be an auto-enzyme in intestinal cell which causes deamination of the glutamine residues in gluten. The deaminated residues then serves as the epitope for CD4+ T-cell recognition and lead to transamidation [11]. These activated T-lymphocytes create an inflammatory response that activates T-helper cells, resulting in a clonal expansion of B-lymphocytes. B-lymphocytes secrete anti-gliadin and anti-tissue-transglutaminase antibodies, which are recognized markers involved in the diagnosis of CD (Figure 1) [2].

The development of antibodies against gliadin in CD can also be attributed to the molecular mimicry mechanism. Several studies found that T-cell response against gliadin was related to prior viral or bacterial infection [12]. The structure of the HLA-DQ2.5–glia-α2 complex antibodies is similar to TCR–HLADQ2.5–P.aeru-α2a complex [12]. Anti-tissue transglutaminase (Anti-tTG) antibodies have a similar structure to Rotavirus VP-7. A study found that all patients with CD had IgG antibodies against rotavirus, while only 32% of control were positive for these antibodies. This explains that the susceptible patients who get infected by Rotavirus tend to develop CD due to molecular mimicry. It was also found that anti-rotavirus VP-7 peptide antibodies cross-react to Toll-like receptor4 (TLR4), desmoglein, and celiac peptide. Since tTG antibodies are similar to VP-7 antibodies, they may lead to systemic symptoms in CD [13]. Additionally, anti-gliadin 1B antibodies show reactivity against jejunal epithelial mucosa cells, especially against brush borders. However, there is no affinity for the crypts [14,15,16].

### 1.3. Extraintestinal Manifestations

The discovery of genetic (HLA DQ2/DQ8) and autoantigen (tissue transglutaminase tTG) serology has led to a steady rise in CD diagnosis over the last few decades, although most patients remain undiagnosed [17]. Clinicians need a high index of suspicion for diagnosing CD as patients can frequently present with nonspecific or extraintestinal manifestations (EIM). Without typical GI symptoms and insufficient knowledge of the EIM, diagnosis is often incorrect or delayed. An adult cohort study showed a mean time of 2.3 months to diagnosis in patients with GI symptoms, compared to a 42-month (3.5 years) delay without [18].

EIMs have been associated with all organ systems, some more common than others. Musculoskeletal manifestations are a common but variable presentation among CD patients. Osteoporosis was the most variable ranging from 26–72% in prevalence and theorized to be from malabsorption (vitamin D deficiency leading to secondary hypoparathyroidism), or chronic inflammation both leading to increased RANKL secretion and osteoclast activity [19,20]. Arthralgia and arthritis have also been reported in 20% to 30% of patients at the time of diagnosis [21].

A frequent dermatologic EIM is the presentation of dermatitis herpetiformis (DH). DH is a chronic pruritic papulovesicular lesions characterized histologically by subepidermal granular IgA deposition. It is commonly associated with the HLA-DQ2 or HLA-DQ8 with approximately 85% Caucasians with DH carrying the HLA-DQ2 [22]. Previous cohort studies showed a prevalence of DH to be 17–20% in untreated CD [23,24,25], however, in 2017, a 10-year cohort study in Finland showed a prevalence of only 4% [26]. Other dermatologic associations can include psoriasis [27], urticaria [28], leukocytoclastic vasculitis [29], alopecia areata, atopic dermatitis and hereditary angioneurotic oedema [30].

Common reproductive complications of CD include amenorrhea, delayed and early menopause [31,32], infertility [33], recurrent miscarriages, intrauterine growth restriction [34,35], and preterm deliveries [36].

There is one known pulmonary co-occurrence of CD and idiopathic pulmonary hemosiderosis, known as Lane-Hamilton Syndrome [37].

Other rare associated conditions can include ischemic heart disease [38], atrial fibrillation [39], dilated cardiomyopathy [40], ocular retinopathy, cataracts [41], uveitis [42], type 1 diabetes mellitus [43], Hashimoto’s thyroiditis, Grave’s disease [44], Addison’s disease [45], hepatic autoimmune diseases (autoimmune hepatitis [46,47,48,49,50], primary biliary cholangitis [51], primary sclerosing cholangitis [44]), and chromosomal abnormalities (Down [52,53,54], Turner [55], and William’s [56] syndromes).

Thus, CD being a multisystem disorder, neurological manifestations are no exception. This review aims to update the current literature on the neurological presentations of CD, treatments for CD and its associated neurological manifestations [57].

## 2. Neurological Manifestations of Celiac Disease

### 2.1. Introduction

**Figure 2:**
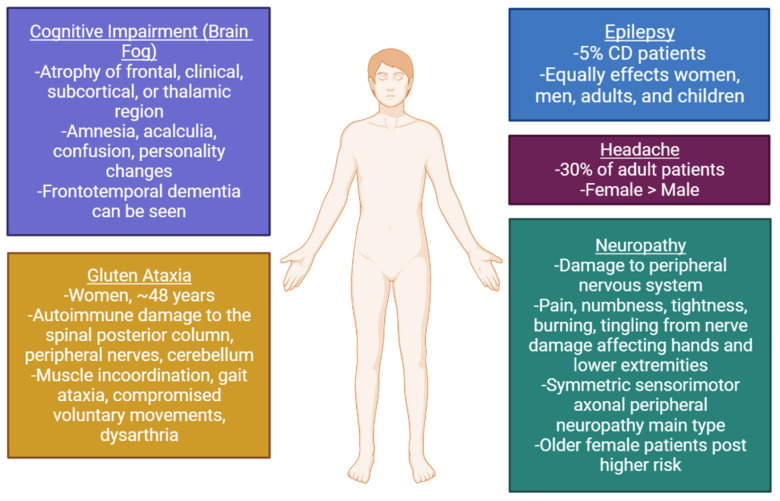
Summary of the neurological manifestations of Celiac Disease.

Although CD primarily affects the GI system, CD is multi-systemic and involves multiple organs, including the skin [58], pancreas [59], thyroid [60], liver [61], heart [62], muscles, bones [63], joints, central nervous system, and peripheral nervous system [64]. Given the diversity of organ involvement in this disease, a precise understanding of each group is important in the diagnosis and management of this condition. Numerous neurological syndromes have been described in association with CD such as ataxia, cognitive impairment, epilepsy, headache, and peripheral neuropathy (Figure 2). Rarely CD can present with myoclonus, internuclear ophthalmoplegia, multifocal leukoencephalopathy, and dementia [65,66].

### 2.2. Gluten Ataxia

Gluten ataxia (GA) is characterized by gluten-sensitivity autoimmunity and is the most common manifestation of CD, however, a presence or lack thereof for an enteropathy is not a pre-requisite for diagnosis [67,68]. GA mostly affects women with a mean age of 48 years [67]. Gluten ingestion causes autoimmune damage to the posterior column of the spinal cord, peripheral nerves, and cerebellum, which are responsible for muscle coordination and gait, leading to compromised voluntary movements [69,70]. Symptoms are usually insidious and chronic in nature, with possible associations to palatal myoclonus, segmental myoclonus, or sensorimotor neuropathy [69,70]. Almost all patients with GA clinically present with gait ataxia or postural deficits. Additionally, up to 70% of patients have dysarthria, nystagmus, and limb ataxia [71].

Digested gluten peptides cross-link and are deaminated by transglutaminase 2, forming an immunostimulatory epitope for HLA-DQ2/DQ8 on antigen-presenting cells [67]. Epitopes are then presented to CD4+ T-cells, which release cytokines to begin the production of antibodies, specifically against gliadin and transglutaminase. Anti-transglutaminase antibodies target tissue transglutaminase TG2, which is ubiquitous, and TG6, which is found richly in the central nervous system and is associated with neuronal differentiation [72]. Research has suggested that anti-TG2 antibodies induce mitochondrial-dependent neuron apoptosis [73].

HLA-DQ2 is detected in approximately 70% of patients, with IgG and IgA anti-gliadin, anti-TG6, and anti-transglutaminase 2 antibodies being detected in the serum and cerebrospinal fluid of patients diagnosed with GA [67]. In rare cases, oligoclonal bands are detected in CSF. 10% of patients have detected HLA-DQ8/DR4 [69,70]. Anti-TG6 is the most specific antibody for the patient’s demonstrating neurological manifestations of GA [74].

The most effective and first-line therapy for GA is a gluten-free diet (GFD) [75]. Strict GFD with serological evidence of antibody elimination appears to stabilize and cause a partial reversal of immune-mediated damage to the CNS [68]. However, improvement of ataxia is not guaranteed, as cerebellar atrophy and loss of Purkinje cells are not reversible. Immunosuppression and IV immunoglobulins can be considered in patients who have strictly followed a GFD for at least 1 year with no improvement in symptoms, as neurological benefits of GFD may not manifest until after 1 year of its introduction, or if ataxia continues to progress [68,75].

### 2.3. Epilepsy

Epilepsy is present in 5% of adult patients with CD [76]. A nationwide Swedish cohort study with 28,000 patients, showed that there was a 1.4-fold increased risk of epilepsy in patients with CD [77]. The risk of epilepsy is equal in all males, females, adults, and children suffering from CD [77].

Pathogenesis of epilepsy in patients with CD is attributed to varied factors including malabsorption of vitamin B12, local deposition of tTG antibodies leading to impaired function of immune complexes, and vasculitis [78]. In a case study, a patient with CD and epilepsy showed recovery and elimination of seizure episodes just from a GFD and without administration of folate. Further evaluation with indirect immunofluorescence using sera showed antibodies against neuronal tissue and glial cells. These findings demonstrate that epilepsy in CD might be related to immunological factors more than folate deficiency [79]. Further research to determine the cause of epilepsy is needed.

Additionally, CD patients with epilepsy are more susceptible to various calcifications in the occipital or parietal lobes [78,79]. Although this susceptibility is quite common, the pathophysiology behind it is not fully clear [80].

Currently, GFD and folate supplements are widely accepted treatments and have shown promising results in reducing the frequency of seizures [80].

### 2.4. Headache

Headaches are a common complaint in patients with CD. In a study, 30% of patients with CD had chronic headaches [81]. A pooled analysis found that headache is prevalent in 26% of adults and 18.3% of pediatric patients with a diagnosis of CD [82].

Another study in patients with CD found headaches to be significantly more prevalent in females compared to males (71.9% in females vs. 28% in males) [83]. A study showed that 24% of patients presented with an initial symptom of headache which eventually led to their diagnosis of CD [84]. Further, CD was comorbid with tension headache in 52%, migraine without aura in 32.5%, and migraine with aura in 15.4% of patients with CD [84].

Even though associations were seen in many studies between various kinds of headaches and CD, the direct pathophysiology of the correlation is not well understood [81,84]. An increase in pro-inflammatory cytokines is seen in CD patients [85]. Thus, it can be hypothesized that the increased tumor necrosis factor-alpha and interleukin-1 Beta play a role in causing migraines [86].

Nevertheless, after initiating GFD and controlling symptoms of CD, patients with prior history of headaches showed no recurrence of headaches [84,87].

### 2.5. Neuropathy

Gluten neuropathy is an autoimmune manifestation in which gluten ingestion causes damage to the peripheral nervous system, disrupting communication between the central nervous system to the body [66]. This is the second most common neurological manifestation, after gluten ataxia [88]. It presents with pain, numbness, tightness, burning and tingling from nerve damage, that initially affects the hands and lower extremities [89]. The median age of onset is 55 years. Estimates of the prevalence of gluten neuropathy are as high as 39%, with an increased risk in older females [90]. Symmetric sensorimotor axonal peripheral neuropathy is the main type of gluten neuropathy, however sensory ganglionopathy has also been noted in studies [88,91]. Symptoms of gluten neuropathy improve when patients follow a GFD, although it may not be completely reversible [92].

### 2.6. Cognitive Impairment (“Brain Fog”)

Various cognitive impairments are seen in most patients with CD. The most common include amnesia, acalculia, confusion, and personality changes [93,94]. Cognitive impairment is mild during the initial stages of CD and worsens as the disease progresses [95].

Most CD patients with cognitive impairment are also diagnosed with generalized atrophy or atrophy of frontal, cortical, subcortical, or thalamic regions [94]. These atrophies are thought to be caused secondary to CD [93,94]. Additionally, patients can present with various dementias such as Alzheimer’s, vascular dementia, and frontotemporal dementia [93].

Since CD enteropathy leads to malabsorption and deficiency of vitamin B12, vitamin E, and folate, they are hypothesized to cause cognitive impairment [96]. However, providing vitamin supplementation does not lead to any improvement in cognitive function [94]. Hence, the pathophysiology leading to cognitive impairment remains unclear [96]. Increased concentration of cytokines in CD patients causes cognitive impairment in different disease processes. One can speculate that these pro-inflammatory cytokines bind and embed in the blood–brain barrier and increase the movement of white blood cells to the brain leading to cognitive deficits [85].

Lastly, brain fog has also been reported in CD patients. It presents with difficulty in finding words, initiating new movements, and staying concentrated, oriented, and attentive. Brain fog completely subsides with the GFD and reappears with gluten exposure. A study found that GFD led to improvement or reduction in all cognitive symptoms in a few patients [94].

## 3. Treatment of Celiac Disease

### 3.1. Introduction

Currently, the only clinically accepted form of treatment for CD is life-long adherence to a GFD [97]. This treatment is safe and effective for most patients [98]. However, there is a high risk of gluten contamination as well as social-economic barriers which hinder long-term maintenance [99].

A 2006 survey conducted on 2681 adult members of the Canadian Celiac Association showed that 44% of participants found difficulties following the GFD which included: determining if foods were gluten-free (85%), finding gluten-free foods in stores (83%), avoiding restaurants (79%), and avoiding travel (38%) [100]. Although the US FDA and Codex Alimentarius, a consensus of international standards for food safety, established the labeling of products “gluten-free” must have a limit of quantitative measurement <20 parts per million (ppm) [101], there is no true consensus of the threshold for triggering symptoms due to individual variability [102]. CD patients need alternative therapy to avoid damage and reduce involuntary or voluntary dietary transgressions; but until new adjuncts and preventative strategies are approved, the GFD will continue to be the mainstay of treatment [103].

Patients with CD need to be followed up closely in outpatient clinics to review dietary compliance, comorbidities and quality of life [104]. Additionally, routine laboratory tests should assess micronutrient deficiencies and cardiovascular risk factors. It is advisable to follow up patients with CD yearly [104].

Currently, there are several non-dietary therapies (NDT) in clinical trials. The NDTs of current interest include tight junction integrity (larazotide acetate) [105], oral enzyme supplements (latiguluinase) [106], TG2 inhibition [107], and induction of gluten tolerance with Nexvax2 vaccine [108].

### 3.2. Tight Junction Integrity

Larazotide acetate (LA), (formerly AT-1001) is a highly polar octapeptide derived structurally from the zonula occludens toxin secreted by *Vibrio cholerae* [109]. It functions as an anti-zonulin receptor inhibitor that minimizes intestinal barrier permeability in and around tight junctions.

LA is also found to activate cytoskeletal actin filaments, promoting tight junction assembly, as well as the active suppression of myosin light chain kinase, stimulating tight junction closure [110].

In a 2021 systematic review and meta-analysis of randomized controlled trials on LA, included four studies comprising 626 patients (465 received LA and 161 given placebo) advocated a good safety profile and superiority of Larazotide over placebo in alleviating GI symptoms in gluten-challenged patients with CD [105]. This study, as well as previous clinical trials [111,112,113,114], show that LA significantly reduces GI symptoms, but there are variable results in measured intestinal permeability assessed with urinary lactulose-to-mannitol (LAMA).

Another study using gluten challenging showed a 70% increase in intestinal permeability of the placebo group versus no increase in patients treated with LA [111]. Placebo patients also reported increased GI symptoms compared to a decrease with LA.

Other clinical trials measuring permeability with LAMA during gluten challenge showed no significant protection versus placebo. This could be due to the high variability of LAMA assessment in outpatient settings [109]. Treatment in these studies also showed an inverse dose effect, with lower doses being more efficacious than higher doses [109]. This relationship has been hypothesized to be due to desensitization of receptors or aggregation of the drug.

A randomized controlled trial showed the treatment dose of 0.5 mg Larazotide significantly decreased not only GI symptoms, but also EIM including headache and tiredness [113].

### 3.3. Oral Enzyme Therapies

Oral enzyme therapies are showing great promise in alternative therapies for CD. Glutenases, gluten-degrading enzymes that are proline & glutamine-specific endoproteases, could be most useful in ameliorating gluten-induced effects from accidental ingestion [115]. Gluten-specific peptidases have been identified in humans, bacteria, fungi, plants, and several insect species [116]. The peptidases must demonstrate stability in the GI tract (low pH, resistance to proteolytic degradation), ability to degrade the immunogenic peptides present in gluten, and not have a damaging effect in the patient [117]. The potential synergism between glutenases that differ in their cleavage specificities and optimum pH values increases the likelihood the mixture would more effectively eliminate the antigenicity of ingested gluten fractions before peptides reach the duodenal lumen [116].

One of the most investigated enzymes is Latiguluinase, also known as ALV003, a gluten-specific protease from an Ep-B2 cysteine endopeptidase from the endosperm of germinating barley, and a prolyl endopeptidase (PEP) from *Sphingomonas capsulate* that is stable and orally active in the stomach [118]. Latiguluinase targets gliadin and degrades it into small fragments in the stomach before passing into the duodenum and damaging intestinal mucosa. A study by Lahdeaho et al. showed ALV003 attenuated gluten-induced small intestinal mucosal injury with a daily dose of 2g gluten [106]. However, a recent phase 2b study by Murray et al. showed that ALV003 did not improve histologic and symptoms scores in 494 CD patients with moderate to severe symptoms versus placebo [119]. A post hoc analysis found that patients who were seropositive receiving the highest dose of ALV003 showed symptomatic improvement in bloating and tiredness [120].

A newly engineered glutenase, Kuma030, seems to be more efficient than ALV003. With a decreased concentration ratio of enzyme:gluten, the 1:100 weight/weight (*w*/*w*) of Kuma30 reduced the immunogenic gluten present in gastric conditions by >99.9%, versus the 1:25 *w*/*w* ratio of ALV003 degrading gluten by 84.4% [121]. While administration of glutenases will likely not replace a GFD, the amplified degradation level of Kuma30 shows promise to provide additional protection if large amounts of gluten were ingested.

### 3.4. Transglutaminase 2 Inhibition

As previously indicated, Transglutaminase has proved to be a pivotal factor in the pathogenesis of CD. Thus, transglutaminase-2 (TG-2) inhibition could prevent the presentation of peptides by HLA-DQ2/DQ8 and reduce the proliferative response of gluten-reactive T-cells [122].

The most well studied are the irreversible inhibitors, such as the ZED1227. ZED1227 is specific, orally active, and targeted against small intestinal mucosa. Results published in July 2021 showed a successful demonstration of its efficacy, safety, and tolerability [107]. Zedira biotech initiated their phase 2b placebo-controlled dose-finding study in November 2021 [107]. The reversible TG-2 inhibitors compete with other natural amine substrates, making the enzyme unavailable to act on gliadin.

Mercaptamine, or cystamine, is the only other commercially available TG2 inhibitor but research has not been implemented for the therapeutic role in CD [123,124].

Interestingly, disulfiram, a drug approved by the FDA for alcohol abuse has the same mechanism as cystamine, and is the first clinically approved compound to show human tTG inhibitory activity [125]. While not approved for CD, further investigations should be pursued for potential therapeutic use.

Badarau et al. described reversible inhibitors as useful for in vitro studies, but noted that in vivo they have the potential to elicit an autoimmune response [126]. This should be considered as well in further studies.

### 3.5. Immunotherapy and Vaccines

Immunotherapy techniques are currently being developed to reduce the inflammatory reaction to gluten consumption in patient with CD. The goal of these immunotherapeutic treatments is to achieve complete tolerance to ingested gluten by targeting CD4+ T-cells in the intestine, by inactivating them when exposed to gluten [127].

Vaccines are being developed to help treat CD, by using the mechanism of action. NexVax2 is an epitope-specific vaccine that helps silence the inflammatory cascade in HLA-DQ2.5 genotype patients [108].

The NexVax2 vaccine contains synthetic peptides of amino acids that include five of the gluten peptides that are most prevalent in HLA-DQ2.5 CD: DQ2.5-glia-α1, DQ2.5-glia-α2, DQ2.5-glia-ω1, DQ2.5-glia-ω2, and DQ2.5-hor3. By introducing synthetic peptides that are involved in the inflammatory process of CD, it assists in tolerating inflammatory T cells specific to these and inactivates the reaction that is commonly seen when ingested [127]. By decreasing the inflammatory reaction, such as the production of IFN-γ, neurological manifestations of CD can be treated or prevented.

Copolymers of sodium 4-styrene sulfonate (SS) and hydroxyethyl methacrylate (HEMA) are being investigated for treatment of CD. These molecules sequester of alpha-gliadin leading to a complex formation [128].

### 3.6. Treatment for Neurological Manifestations of Celiac Disease

Other studies have been done to improve for example, functioning of Purkinje cell by drugs such as, Riluzole, Troriluzole, CAD-1883, and TAK-831 Riluzole [129]. These act by promoting the opening of small-conductance calcium-activated potassium channel opener and enhancing glutamate transporters. Troriluzole is a pro-drug of Riluzole and is currently in phase 3 trial focusing on spinocerebellar ataxia treatment [130]. CAD-1883 is an allosteric modulator of small-conductance calcium activated-potassium channels. TAK-831 functions by increasing plasma, CSF, and cerebellum levels of D-serine, by inhibiting D-amino acid oxidase (DAO). Increased levels of D-serine in these regions, which act on glutamate receptors leading to regulation of the cerebellar output [129]. Promotion and enhancement of these work to reduce the over-firing of Purkinje cell fibers, thus reducing symptoms of ataxia. It has not been determined if these drugs can be beneficial for patients with recurring gluten ataxia while on a strict GFD. Many of these drugs are still in clinical trials therefore further research must be undergone to see if there is any benefit from these treatments. Larazotide, an anti-zonulin receptor inhibitor, has shown to reduce headaches and tiredness [113]. However, its effects on potentially reducing the risk of other neurological manifestations remain unclear.

## 4. Conclusions

There has been a steady rise in the diagnosis of CD secondary to increased awareness. The standard treatment of CD is a strict lifelong GFD. However, a significant proportion of individuals with CD have barriers to achieving GFD creating a growing need for novel therapeutics. Newer drugs have shown promise in the initial studies.

It is proven that the best treatment for neurological manifestations is treating the underlying CD by maintaining a GFD. However, at times more severe manifestations, such as ataxia, cannot be resolved by a GFD alone.

Vitamins such as B6, B12, folate and metals such as copper deficiencies due to malabsorption could be potential causes of neuropathy. Nutritional deficiencies, which are rarely the sole cause of neurological manifestations, are easily correctable and should be investigated.

Oral enzyme therapies are a safe group of drugs with the potential to reduce accidental gluten exposure and mucosal injury. Clinical trials have yielded mixed results thus far. However, further studies need to explore the effects of using combinations of oral enzymes.

These therapies improve the mucosal integrity which will potentially reduce the risk of nutritional deficiencies. Additionally, reduced immunologic response leads to a decrease in the number of autoantibodies thereby reducing the risk of autoimmune damage to various organ systems. Combined, these mechanisms could potentially reduce the prevalence and severity of EIMs. Further clinical trials need to be conducted to assess the efficacy of the novel therapeutics as well as GFD on preventing or treating the neurological manifestations of CD.

There is an increased prevalence of individuals presenting with neurological manifestations of CD such as ataxia, cognitive impairment, epilepsy, headache, and neuropathy. However, the underlying pathophysiology of these manifestations remains unclear. A better understanding of the pathophysiology may aid in guiding targeted therapeutics in the future and possibly reversing the neurological dysfunction.

The nonclassical or extraintestinal presentation of CD makes it challenging to diagnose CD. We hope to increase clinician awareness about the neurological manifestations of CD and clinicians should remember to consider CD in individuals presenting with idiopathic neurological dysfunction. Diagnostic testing of CD is recommended in patients presenting with non-specific neurological symptoms who have a strong family history of CD.
